# The Acceptability, Engagement, and Feasibility of Mental Health Apps for Marginalized and Underserved Young People: Systematic Review and Qualitative Study

**DOI:** 10.2196/48964

**Published:** 2024-07-30

**Authors:** Holly Alice Bear, Lara Ayala Nunes, Giovanni Ramos, Tanya Manchanda, Blossom Fernandes, Sophia Chabursky, Sabine Walper, Edward Watkins, Mina Fazel

**Affiliations:** 1 Department of Psychiatry University of Oxford Oxford United Kingdom; 2 Department of Psychological Science University of California Irvine, CA United States; 3 German Youth Institute Munich Germany; 4 School of Psychology University of Exeter Exeter United Kingdom

**Keywords:** adolescent mental health, marginalized groups, smartphone apps, engagement, implementation science, mobile app, smartphone, mobile health, mHealth, mental health, challenges, acceptability, young, effectiveness, mobile phone

## Abstract

**Background:**

Smartphone apps may provide an opportunity to deliver mental health resources and interventions in a scalable and cost-effective manner. However, young people from marginalized and underserved groups face numerous and unique challenges to accessing, engaging with, and benefiting from these apps.

**Objective:**

This study aims to better understand the acceptability (ie, perceived usefulness and satisfaction with an app) and feasibility (ie, the extent to which an app was successfully used) of mental health apps for underserved young people. A secondary aim was to establish whether adaptations can be made to increase the accessibility and inclusivity of apps for these groups.

**Methods:**

We conducted 2 sequential studies, consisting of a systematic literature review of mental health apps for underserved populations followed by a qualitative study with underserved young male participants (n=20; age: mean 19). Following the PRISMA (Preferred Reporting Items for Systematic Reviews and Meta-Analyses) guidelines, an electronic search of 5 databases was conducted in 2021. The search yielded 18,687 results, of which 14 articles met the eligibility criteria.

**Results:**

The included studies comprised a range of groups, including those affected by homelessness, having physical health conditions, living in low- and middle-income countries, and those with sexual and gender minority identities. Establishing and maintaining user engagement was a pervasive challenge across mental health apps and populations, and dropout was a reported problem among nearly all the included studies. Positive subjective reports of usability, satisfaction, and acceptability were insufficient to determine users’ objective engagement.

**Conclusions:**

Despite the significant amount of funding directed to the development of mental health apps, juxtaposed with only limited empirical evidence to support their effectiveness, few apps have been deliberately developed or adapted to meet the heterogeneous needs of marginalized and underserved young people. Before mental health apps are scaled up, a greater understanding is needed of the types of services that more at-risk young people and those in limited-resource settings prefer (eg, standard vs digital) followed by more rigorous and consistent demonstrations of acceptability, effectiveness, and cost-effectiveness. Adopting an iterative participatory approach by involving young people in the development and evaluation process is an essential step in enhancing the adoption of any intervention, including apps, in “real-world” settings and will support future implementation and sustainability efforts to ensure that marginalized and underserved groups are reached.

**Trial Registration:**

PROSPERO CRD42021254241; https://www.crd.york.ac.uk/prospero/display_record.php?RecordID=254241

## Introduction

### Background

Addressing health inequities is a key challenge for the mental health field, especially when trying to ensure that interventions and services are accessible and acceptable for all populations. Nearly 50% of lifelong mental health disorders begin by the age of 14 years, and by the age of 24 years, 75% of mental health disorders have begun [1]. Given the frequent onset of mental health problems during youth, here defined as the period between 15 and 24 years, special attention must be paid to older adolescents, including those from underserved and marginalized minority groups and socioeconomically deprived backgrounds [2]. In these groups, common barriers to accessing mental health services can be exacerbated (eg, poor mental health literacy, lack of knowledge about where to seek help, negative attitudes toward professional help seeking, embarrassment, preference for self-reliance, fear of stigma, and confidentiality concerns), and additional barriers exist (eg, reliance on informal supports, shame, lack of housing or money, and therapist factors, such as different race and level of experience), creating increased risk of untreated mental health problems and thus poorer mental health outcomes [3-7]. In this study, marginalized and underserved populations are defined as those with higher prevalence of mental health problems and lower rates of help seeking, such as racially and ethnically minoritized individuals, rural and remote communities, financially deprived groups, individuals experiencing homelessness, refugee and migrant populations, and sexual and gender minority groups [8-10] and those with lower inclusion in mental health intervention research than one would expect from population estimates [11], respectively. These groups are exposed to risk factors for poor mental health and experience disparities in mental health care, including lower access to care, poorer treatment quality, and limited engagement in treatment [5,10,12].

Smartphone apps could offer an opportunity to deliver mental health and well-being resources and interventions in a scalable, cost-effective, and potentially personalized manner, particularly for those who experience the greatest barriers to accessing health care [13,14]. Given that smartphone ownership is nearly ubiquitous among young people in high-income nations and increasingly across lower-resource settings, apps have the potential to address some of the accessibility issues in service provision for young people’s mental health [15]. Young people are more digitally connected (ie, they are more likely to own smartphones and spend more time on the web) and more likely to seek health information on the web than older generations, meaning that app-based interventions may be particularly well-suited for this population [16,17].

Not surprisingly, the number of mental health apps being developed, both commercially and in academic research programs, has expanded rapidly, outpacing scientific evaluations of their effectiveness [18,19]. Emerging evidence suggests that some apps may produce significant symptom improvement across multiple outcomes, compared with waitlist or control conditions [20-22]. Despite promise, empirical research often fails to translate into meaningful and sustained implementation in “real-world” settings [23,24]. Research has focused primarily on efficacy under ideal “laboratory” conditions rather than effectiveness in real-world settings [25]. Therefore, at present, most apps, especially those available to the public, lack strong empirical support [19]. The acceptability (ie, perception that a given technology is useful, agreeable, palatable, or satisfactory); accessibility (ie, the technology being easy to obtain or use); engagement (ie, initial adoption and sustained interactions with the technology, including the level of app use, intervention adherence, and premature dropout); and feasibility (ie, the actual fit, utility, or suitability and the extent to which the technology can be successfully used or conducted within a given context) of apps for marginalized and underserved groups remain poorly understood [25-28]. Although mental health apps may provide a possible solution, marginalized and underserved groups of young people face unique challenges to engage with and benefit from these interventions (eg, intervention cost, content that is not culturally attuned and lack of reliable access to the internet) and are typically underrepresented in intervention research [25,27]. Although increased access is often seen as a major benefit of digital mental health interventions, issues related to the “digital divide” describe the phenomena that technology is not equally available to all social groups due to economic, social, or cultural inequalities and is a potential ethical concern [29]. Furthermore, underrepresentation in the intervention development process potentially reinforces structural inequalities by limiting the availability of products that are culturally accessible, inclusive, and effective or by skewing the product features to attract young people from more advantaged backgrounds [9,14]. Therefore, considering diversity, equity, and inclusion issues at the outset of health care research as well as within app evaluation is essential to prevent the perpetuation of existing inequities [27].

To date, little has been published on the attempts to create new or adapt existing app interventions to meet the heterogeneous needs of diverse groups of young people [9,14]. Moving forward, careful consideration is needed to ensure optimal leveraging of all mental health intervention research, including that of mental health apps, to increase health equity while also ensuring that innovations do not inadvertently widen the digital divide and exacerbate health inequalities [14]. Although the efficacy of many mental health apps remains unclear, future attempts to translate findings for underserved populations will need to ensure that all apps are developed with enough flexibility to fit a wider range of user needs and preferences. To achieve this goal, research is needed to assess the acceptability and feasibility of mental health apps for underserved young people to ensure that they are not further excluded from research and to advance toward mental health provision that meets their needs.

### This Research

We conducted two sequential studies: (1) a systematic literature review and (2) a qualitative study with a targeted sample of young people who often are underrepresented in research, with limited access to health care and socioeconomic deprivation. The overarching aim of these combined studies was to better understand whether mental health apps are feasible and acceptable to underserved young people. A secondary aim was to determine which adaptations might enable accessibility of and effective engagement with mental health apps for these groups.

The research questions of interest were as follows: (1) On the basis of the existing literature, are mental health apps acceptable, feasible, and engaging for marginalized and underserved young people and how have these constructs been measured? (2) On the basis of the qualitative study, what are young people’s experiences of using a mental well-being app, including its acceptability, feasibility, and level of engagement? (3) On the basis of both studies, are apps an acceptable, feasible, and engaging intervention approach to meet the specific needs of underserved young people? What adaptations can be made to ensure that mental health apps are accessible and inclusive for these groups?

## Methods

### Overview

To fully address our research questions, we adopted a 2-pronged approach. First, we conducted a systematic review of the literature to better understand the acceptability and feasibility of mental health apps for underserved young people. To explore the findings of the systematic review in greater depth and to provide further insights from multiple perspectives, we next conducted a qualitative study with young men not in education, employment, or training (NEET) in the United Kingdom and Spain and asylum seekers and refugees in Germany. The interviews were conducted between August 2021 and February 2022.

### Systematic Review

#### Literature Search and Search Strategy

An electronic literature search was performed in English on the following databases from January 2009 to May 2021: Cochrane Library, Embase, MEDLINE, and PsycINFO. We used key search terms relating to (1) underserved young people; (2) mental health mobile apps; and (3) acceptability, feasibility, and engagement. The search strategy was guided by similar reviews exploring digital mental health interventions for young people [25,30], and the terms for apps were derived from Cochrane reviews [31,32]. An updated search was conducted in September 2023. The full search strategies are available in Multimedia Appendix 1.

#### Inclusion and Exclusion Criteria

Screened articles were included if (1) the study targeted marginalized and underserved young people with a mean age of 15 to 25 years, including individuals who were NEET, apprentices, teenage parents, members of minoritized racial or ethnic groups, members of sexual and gender minoritized groups, residents of low- and middle-income countries (LMICs), experiencing homelessness, socioeconomic deprivation, refugees or asylum seekers, and migrants; individuals with substance use disorders; those under state or statutory care; people with physical disabilities; and individuals involved in the criminal justice system or incarcerated; (2) the intervention was a “native” mobile app (ie, not on a web browser), whose primary aim was to promote well-being, prevent mental health problems, or treat existing mental health problems, delivered as a stand-alone intervention or as an adjunct to therapist-assisted interventions; (3) the primary outcome was a measure of mental health or well-being; and (4) the study reported a measure of user acceptability or feasibility.

Articles were excluded if (1) the mean age of participants was outside of the 15 to 25 years range; (2) the intervention was not a mobile app, that is, other digital interventions, including teletherapy (eg, therapy delivered by phone, SMS text messages, video platforms, or PCs); and (3) there was no measure of acceptability or feasibility. Gray literature was not included in the search.

#### Study Selection

**Figure 1:**
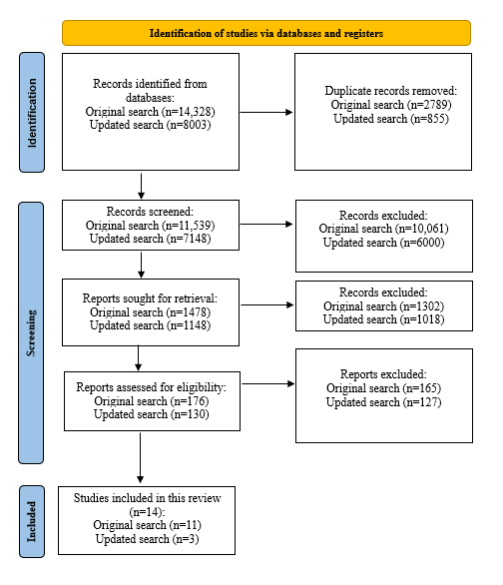
PRISMA (Preferred Reporting Items for Systematic Reviews and Meta-Analyses) flowchart of the study selection process.

In accordance with the PRISMA (Preferred Reporting Items for Systematic Reviews and Meta-Analyses) guidelines [33], the flowchart presented in Figure 1 provides step-by-step details of the study selection procedure. The PRISMA checklist is provided in Multimedia Appendix 1. The search strategy identified 11,539 citations after deduplication. After an initial screening of the titles, which resulted in the exclusion of 10,061 (87.19%) irrelevant entries, the abstracts of 1478 (12.81%) studies were screened by 4 members of the review team (LAN, HAB, BF, and TM). The identified 176 (11.91%) full texts were then screened by LAN. In this final stage, 11 (6.2%) studies, corresponding to 9 interventions, were identified for inclusion in the review, with 8 (73%) found through the electronic search and 3 (27%) through manual searches of the reference lists of relevant articles. The updated search identified a further 7148 citations after deduplication, of which 3 met inclusion criteria and were included in the review.

#### Data Extraction

Data were extracted by 1 reviewer (LAN, HAB, or BF) and reviewed for accuracy and completeness by a second reviewer. After verifying all the extracted data, discrepancies were resolved by discussion or adjudication by another author (MF). Extracted data included information on study characteristics (ie, authors, publication year, country, study design, and study population); intervention characteristics (ie, characteristics of the technology, app name, therapeutic modality, and intervention outcomes); and feasibility and acceptability.

#### Quality Assessment

We used the Mixed Methods Appraisal Tool (MMAT; version 2018) to assess the methodological quality of the included studies [34]. MMAT was developed by combining the core relevant methodological criteria found in different well-known and widely used qualitative and quantitative critical appraisal tools. The MMAT consists of 2 screening questions applicable to all types of study design and a further 5 questions applicable to specific study designs. Responses were rated on a categorical scale as “no,” “unclear,” or “yes” to any of the methodological quality criteria. Quality assessments were made by 1 reviewer (TM). We did not exclude any studies based on quality assessment scores.

#### Data Synthesis and Analysis

The extracted data were collated and summarized to produce a narrative summary of the study; sample characteristics; and acceptability, feasibility, and engagement outcomes. A codebook approach was used to code and synthesize implementation data from all available sources according to the outcome categories [35].

### Qualitative Study

#### Study Context

The Emotional Competence for Well-Being (ECoWeB) cohort multiple randomized controlled trial involved a longitudinal prospective cohort to examine the well-being, mental health, and emotional competence in individuals aged 16 to 22 years across 12 months. The experimental arm was an emotional competence self-help app (ie, MyMoodCoach).

#### Intervention

MyMoodCoach was designed to test if an app could improve different processes affecting mental well-being, including, but not limited to, improving emotion regulation by reducing maladaptive strategies such as worry and rumination and replacing them with constructive alternatives and problem-solving and enhancing emotional knowledge and perception through psychoeducation and learning tasks. The app was designed for young people broadly and was not targeted at a specific population. Full details of the ECoWeB trial are reported in the study protocol [36].

#### Participant Recruitment

In parallel to the trial, we additionally recruited young people to understand the views and experiences of those underrepresented in the study sample (and most other app-based studies). As there was an overrecruitment of White, university-educated female participants in the study sample, we decided to only recruit male participants and to focus on 2 specific groups: NEET men and migrant populations (including both voluntary and forced migrants). In the United Kingdom and Spain, recruitment was conducted through a variety of channels, including Twitter, targeted adverts on social media (eg, Instagram and Facebook), newsletters sent to youth and practitioner networks, outreach to third sector organizations and mental health support groups, and advertisements placed on university and charity websites. In Germany, participants were recruited directly from refugee homes, integration courses, migration services, and youth centers.

#### Inclusion and Exclusion Criteria

Inclusion criteria required the participants to be (1) aged between 16 and 22 years; (2) able to speak and read English, Spanish, or German; (3) male; (4) NEET (United Kingdom), NEET or migrant (Spain), or asylum seeker or refugee (Germany); (5) having access to a smartphone with the minimal technological specifications necessary for the app (ie, iOS 9 or later or Android 8.0 or later); and (6) having access to the internet via mobile data or Wi-Fi. The exclusion criteria were having current suicidal ideation, psychosis, or bipolar disorder.

#### Procedure

The research team was led by the principal investigator, MF, professor of Adolescent Psychiatry and Consultant in Children’s Psychological Medicine, with particular expertise in the mental health needs of refugee populations. EW, professor of Experimental and Applied Clinical Psychology, Chartered Clinical Psychologist, provided additional supervisory input and guidance as a leading expert in the field of child and adolescent mental health research. SW, professor of Education with a focus on youth research and director at German Youth Institute, provided supervisory oversight of the interviews in Germany. HAB and LAN were postdoctoral researchers at the time the research was conducted, both of whom have several years of experience conducting qualitative research with young people and expertise in analyzing qualitative data. SC, researcher at the German Youth Institute, conducted and analyzed the interviews in Germany and has experience conducting qualitative research with refugee populations. The authors had no relationship with any of the participants.

In the United Kingdom and Spain, participation involved (1) short questionnaires about mood and feelings and demographic questions such as age, gender, race, ethnicity, educational attainment, and country of origin; (2) downloading MyMoodCoach and using it for 4 weeks; and (3) completing a follow-up interview. Interviews were conducted by HAB and LAN via MS Teams (Microsoft, Corp) and lasted approximately 45 minutes. A similar procedure was followed in conducting the interviews with refugees in Germany, but given the likely language barriers in navigating the app, an additional, earlier interview was included 2 to 3 weeks after the initial instructions had been sent to explore if features of the app were understood and to clarify questions. After 2 further weeks, a second interview was conducted. One interview was conducted in person, while all the others were conducted over the telephone.

Interviews followed a semistructured schedule based on a taxonomy of implementation constructs [26]. Topics included self-reported app use, satisfaction and feedback about content, usability, and acceptability (Multimedia Appendix 2). In Germany, the interview topic guide was translated and adapted to the target group. As not to stigmatize the young men, the wording “refugee” was avoided, and instead, when referring to the target group, the wording “young man such as yourself” was used.

### Ethical Considerations

In the United Kingdom, ethical approval was granted by the University of Exeter Research Ethics Committee (eCLESPsy000048 v10.0); in Spain, by the Jaime I University Research Ethics Committee (CD/93/2021); and in Germany, by the Ethics Committee of the Faculty of Medicine at Ludwig-Maximilians University Munich (PNr 19-0468/19-0315). Cognizant of the ethical and practical implications of conducting research with underserved populations, we conducted the interviews in private, quiet spaces and in a friendly and reassuring manner to ensure that participants felt comfortable and safe.

With prior consent, all interviews were audio recorded. Participants were reimbursed with a shopping voucher of up to €50 or £50 (US $64) for their time and app use.

### Analytic Strategy

Interviews were transcribed verbatim, and the transcripts were assigned a unique pseudonym to anonymize participants. The interviews were analyzed using a combination of theory- and data-driven analysis techniques, consisting primarily of deductive, theory-driven thematic analysis [37]. Analysis of the transcripts in the United Kingdom and Spain was conducted by HAB, using NVivo (version 11; Lumivero). Following a similar procedure, the interviews in Germany were transcribed and analyzed using the coding software MAXQDA (VERBI GmbH) by SC.

Initial familiarization with the data was achieved through the transcription process and iterative rereading of the interviews. Analysis was carried out through a recursive process of open coding, when concepts were named and their properties and dimensions identified, followed by axial coding, when links and associations were drawn between codes. Codes were based on language used by the young people and were applied to each new unit of meaning. Data extracts were multiply coded when appropriate, as were contradictory and minority features of the data. The data set was iteratively reviewed, and codes were systematically applied to the whole data set until a finalized coding manual was established. Codes were organized into potential themes using thematic maps and tables. The development of the coding manual was iteratively reviewed and refined through discussion with all authors throughout the analysis process to ensure the reliability and rigor of the process and results.

### Approach to Inquiry

Analysis was conducted from a critical realist perspective to provide a more nuanced understanding and explanation of participants’ experiences [38]. This position assumes that although participants’ accounts provide important insights about the real world, these accounts are not objective and represent an interpretation of reality [39]. These data require interpretation and explanation by the researcher, who also has their own perspectives on the world, to better understand the underlying mechanisms and processes and, in turn, make recommendations for practice [40].

## Results

### Systematic Review

#### Study Characteristics

Characteristics of the 14 included studies, examining 12 interventions, are presented in Table 1. The included studies were published in the United States (n=3), Australia (n=3), Switzerland (n=2), India (n=2), South Korea (n=1) and Germany (n=1). As for the type of intervention, 7 apps were stand-alone, and the rest (n=5) were delivered in combination with other forms of professional support. The interventions (n=12) were targeted at apprentices and the unemployed (n=3), homeless populations (n=2), those with physical health conditions (n=2), sexual and gender minoritized individuals (n=3), those residing in LMICs (n=1) and those with co-occurring autism spectrum disorders (n=1). Most of the interventions had been co-designed with young people (11/12, 92%). The study sample size ranged from 9 to 877, with 4 of the 14 studies having a sample size of >200 participants.

**Table 1:** Study characteristics.

Study, year	App name	Population	Country	Study design	Intervention focus	Sample size, n	Age (y), mean (SD)	Sex (female, %)
Bohleber et al [41], 2016	Companion App	Employed (apprentices) and unemployed	Switzerland	Mixed methods	Peer mentoring and interactive health content to increase social support and reduce stress	619	Employed: 16.9 (1.73), unemployed: 18.4 (1.96)	Employed first year: 50.2, employed second year: 56.7, unemployed group: 40.4
Deady et al [42], 2020	HeadGear	Apprentices	Australia	Mixed methods	Behavioral activation and mindfulness therapy	54	21.68 (3.62)	4
Fleming et al [43], 2017	TODAY!	Sexual minority male participants	United States	Qualitative	CBT^a^ to manage anxiety and depressive symptoms	9	19. (0.71)	0
Francis et al [44], 2020	CyFi Space	Individuals with CF^b^	Australia	Mixed methods	Social connectedness and well-being of young people living with CF	22	12-17	50
Geirhos et al [45], 2022	Minddistrict. Program: youthCOACH_CD_	Individuals with a chronic illness (CF, JIA^c^, and T1D^d^)	Germany	Pilot RCT^e^	iCBT^f^ targeting symptoms of anxiety and depression	30	16.13 (2.34)	73
Glover et al [46], 2019	A suite of 15 apps including Pocket Helper2.0^g^	Individuals experiencing homelessness	United States	Pilot study	Daily coping skills; focused tips and brief CBT	100	20.03 (1.83)	39
Gonsalves et al [47], 2019	POD Adventures	Students	India	Intervention design	Problem-solving for adolescents at risk of anxiety, depression, and conduct difficulties	Students: 118, service providers: 16	14	46
Gonsalves et al [48], 2021	POD Adventures	Students	India	Pilot study	Problem-solving for adolescents at risk of anxiety, depression, and conduct difficulties	230	15.57	50
Haug et al [49], 2017	ready4life	Vocational students	Switzerland	Pilot study	Life skills training: self-management skills, social skills, and substance use resistance	877	17.4 (2.7)	58.3
Leonard et al [50], 2018	Calm Mom	Mothers experiencing homelessness	United States	Pilot study	Emotion regulation strategies	49	18.54	100
Schueller et al [51], 2019	Pocket Helper, Purple Chill, Slumber Time, and IntelliCare (12 mini apps)^g^	Individuals experiencing homelessness	United States	Pilot study	Emotional support and coping skills	35	19.06 (0.85)	65
Escobar-Viera et al [52]^h^, 2023	REALbot	Rural living LGBTQ+ youth	United States	Pilot study	Chatbot deployed on Facebook Messenger and Instagram apps to deliver educational content	20	16.6 (1.5)	65
Torok et al [53]^h^, 2022	LifeBuoy	Community sample (over 50% of sample LGBQI sexual minority)	Australia	RCT	DBT to treat persistent emotional dysregulation to prevent self-harm and suicidal behaviours	455	21.5 (2.18)	84.6
Yang and Chung [54]^h^, 2022	HARU ASD	Individuals with ASD	South Korea	RCT	CBT for anxiety and co-occurring intellectual disability	30	20.97 (5.06)	10

^a^CBT: cognitive behavioral therapy.

^b^CF: cystic fibrosis.

^c^JIA: juvenile idiopathic arthritis.

^d^T1D: type 1 diabetes.

^e^RCT: randomized controlled trial.

^f^iCBT: internet-based cognitive behavioral therapy.

^g^Mobile phones were preloaded with several apps designed to promote mental health wellness and provide real-time resources. Pocket Helper was 1 app specifically designed for this study.

^h^Studies identified in the updated search.

#### Study Quality

The included studies varied in their methodological quality (Multimedia Appendix 3) [41-54]. Most (13/14, 93%) were judged to contain possible limitations in at least 1 criterion. All studies but 1 (13/14, 93%) were clear in their description of study participants or the process of recruiting a sample representative of the population of interest. All studies addressed the research question using collected data and reported in some way on feasibility and acceptability. Most studies (12/14, 86%) effectively used appropriate qualitative, quantitative, or mixed methods to answer their research question. However, many studies (6/14, 43%) did not have a sufficiently large sample to warrant definitive conclusions about the feasibility and acceptability of the intervention studied.

#### Acceptability and Feasibility

User acceptability was found to be high across all included studies, with participants rating the apps positively and reporting high satisfaction with the content of the interventions (Table 2). In studies where participants were asked to indicate if they would recommend the study to someone else, the vast majority reported they would [42,44-46,51]. It is notable that despite many users across the studies reporting high satisfaction levels and being willing to recommend the apps, they themselves did not intend to continue using the apps (ie, low predicted engagement), as they did not find them useful or relevant for their own circumstances [41,42,51]. It is also important to note that many studies incentivized participation with payments, prize draws, and vouchers, and this may have influenced acceptability ratings and engagement [41-43,46,49-51,53,54]. Furthermore, in the only study that asked if participants would pay to use apps, most were unwilling to do so [42].

**Table 2:** Acceptability, feasibility, engagement, usefulness, accessibility, and outcomes.

Reference, year	App name	Measurement	Acceptability and feasibility	Engagement	Barriers to engagement	Perceived usefulness	Accessibility and inclusivity	Intervention outcomes
Bohleber et al [41], 2016	Companion App	Questionnaires and semistructured interviews	Adolescents regarded the concept of the app as well conceived, especially the peer-mentoring system. However, the app did not compare well to other available apps.	Engagement decreased markedly after the first 2 weeks. Average daily visits: in the first 2 weeks, 61; after 6 months, 8.	Technical problems, unclear benefits, and lack of time.	Content was judged informative and interesting. However, some reported that the purpose of app was not evident.	Unemployed participants suggested that reminders to use the app would help.	No significant effect on stress or the perception of social support.
Deady et al [42], 2020)	HeadGear	Questionnaires, semistructured interviews, and focus groups	Apprentices rated the app positively, (average 4/5 stars). Participants had no or neutral willingness to pay for the app. Most would widely recommend the app but predicted their own use would be infrequent (3-10 times) over the next 12 months.	Users completed approximately one-third of the app challenges.	Noncompletion of challenges attributed to “forgetting” and choosing not to “catch up.” Users wanted to be able to skip challenges and suggested gamification and greater personalization.	87.2% claimed it had at least moderately improved their mental fitness. Moderate impact on awareness, knowledge, attitudes, intention to change, help seeking, and behavior change around mental health and well-being.	Participants emphasized the importance of gamification and greater personalization, for example, through the inclusion of personalized music.	No significant differences between baseline and 3-month follow-up measurements. Engagement (intervention completion) directly related to effectiveness.
Fleming et al [43], 2017	Today!	Semistructured interview	Expressed enthusiasm for a comprehensive mobile phone app designed to treat clinically significant symptoms of anxiety and depression among young sexual minoritized men.	Not assessed in this paper.	Weekly phone calls with the coach was described by participants as a barrier to engagement.	Overall, participants had positive reactions to the app, but each individual found different features to be useful (eg, community resources and mood rater).	Usability testers had a wealth of suggestions for topics they would like to see addressed in this kind of app.	Not assessed in this paper.
Francis et al [44], 2020	CyFi Space	Questionnaire, group, and individual interviews	Acceptability of the app was rated moderate.	Overall, 37% recruitment response rate. 77% participants used the app at least once a week. Some participants indicated that the use of the app declined as the 6-week trial progressed.	40.9% reported watching the entertainment and motivational videos.	Many participants found the app both useful and fun to use and agreed they would. recommend the app to others.	Participants rated the app’s usability as high. Age-related accessibility measured.	Not assessed in this paper.
Geirhos et al [45], 2022	Minddistrict. Program: youthCOACH_CD_	Questionnaires	Content was perceived as appropriate. 58% would recommend intervention to a friend, 17% would likely recommend it, 17% would partly recommend it, and 8% would not recommend it.	Intervention adherence=40%, dropout=20%.	Not reported.	Individual tasks perceived as particularly helpful.	Not explicitly reported.	No symptom improvement. Small sample.
Glover et al [46], 2019	Pocket helper 2.0	Questionnaires	73% would recommend the program.	48% of the sample completed the 3-month assessment, while 19% completed the 6-month assessment.	Use and satisfaction with various features reported.	63% of participants at 3 months and 68% of participants at 6 months reported at least moderate benefit from intervention	Designed for youth experiencing homelessness based on the initial input from these youths and was refined based on the feedback received during a previous pilot trial.	Not assessed in this paper.
Gonsalves et al [47] , 2019	POD^a^ Adventures	Focus group discussions, co-design workshops, and user testing	Service providers highlighted that self-help was not a culturally congruent concept for most Indian adolescents.	Not reported in this paper.	Following user testing, activities were shortened to be kept <2-minutes to minimize boredom and disengagement.	Problem-solving reported as being a useful and valued skill.	Design was sensitive to cultural context, language, participant media preferences, and digital access helped focus on user needs. Adaptations were made to address widespread literacy difficulties.	Not assessed in this paper.
Gonsalves et al [48], 2021	POD Adventures	Questionnaires and semistructured interviews	Satisfaction scores ranged from good to excellent.	Intervention completion rate was 92%.	App generally considered easy to use, but a few participants identified confusing game components and issues related to typing and difficult log-in passwords.	Most participants felt that the program had positively impacted their prioritized problem.	As reported in [47].	Outcomes at 4 weeks showed significant improvements on all measures.
Haug et al [49], 2017	Ready4life	Questionnaires	Large proportion of invited adolescents participated. Program evaluated as “very good” or “good” by 94.6% of participants.	Follow-up assessments were completed by 49.7% of the participants. Of the 39 program activities, the mean number carried out was 15.5. In total, 15% failed to engage in any activity, and 52% engaged in fewer than half of the activities.	Participation in the program was lower in male participants and among those reporting an immigrant background.	Not explicitly reported.	Not explicitly reported.	Statistically significant increases in targeted life skills, decline in at-risk alcohol use, and stable rates for tobacco and cannabis use.
Leonard et al [50], 2018	Calm Mom	Technology logs, questionnaires, and in-depth semistructured interviews	Participants felt the general content of app was highly relevant. 75% were “very” satisfied, and 18% were somewhat satisfied.	Mean of 14.77 minutes of using the app. Participants used at least one of the elements on the app on average on 44% of days when they had the study phone.	Technology challenges.	Supported their ability to effectively regulate their emotions.	Majority of participants noted that the app was very accessible, and several indicated that they felt less alone and felt genuinely cared for.	Not assessed in this paper.
Schueller et al [51], 2019	Pocket Helper, Purple Chill, and Slumber Time	Questionnaires	Satisfaction was high; 100% of participants would recommend the program, and 52% reported that they were very or extremely satisfied app.	57% of the participants completed all 3 sessions. Mean 2.09 sessions.	Mobile phone loss (through damage, theft, or other loss).	43% reported app as helpful; 48% found the skills they learned to be beneficial; 43% regularly used the skills.	The apps were preinstalled on all mobile phones before distribution to participants.	Participants experienced limited change on clinical outcomes with small effect sizes.
Escobar-Viera et al [52]^a^, 2023	REALbot	Questionnaires	High user satisfaction. Acceptability rated 5.3/7 but only 25% of participants described the app as exciting or leading edge.	42% of participants interacted with the app for 2 or more days.	Primary challenges were that app felt robotic and not smart enough.	Usability ratings were high on both measures.	Lack of voiceover feature.	Nonsignificant changes in scores of perceived isolation, depressive symptoms and social media self-efficacy.
Torok et al [53]^a^, 2022	LifeBuoy	Questionnaires	Not reported	71.5% completed 5 or more modules (completers)	Participants who completed first survey had significantly lower baseline anxiety symptoms compared to those who did not complete it.	Not reported	Not reported	Depression, anxiety, distress, and well-being symptoms improved in app group and control.
Yang and Chung [54]^a^, 2022	HARU ASD	Questionnaires	Acceptable scores in the Satisfaction and Usability Questionnaire.	No participants dropped out.	Not reported	Not reported	Not reported	Significant decrease in anxiety level, an increase in positive affect, and a decline in stereotypic behaviors, hyperactivity, noncompliance, and inappropriate speech.

^a^Studies identified in the updated search.

Regarding co-design strategies used in these studies, early stakeholder consultation and service provider focus groups were conducted in the early development phase of POD Adventures, a gamified intervention for people with or at risk of anxiety, depression, and conduct difficulties in India; the results highlighted that “self-help” was not a culturally congruent concept for most Indian adolescents [47]. This early feedback was important as it revealed the norms around seeking or receiving direct instruction from parents, teachers, and other elders and that support from a counselor might be necessary to ensure acceptability, feasibility, and engagement [47]. The app was therefore designed to incorporate a combination of teaching methods, including direct instruction, modeling, and practice to accommodate different learning styles and to emphasize self-efficacy [47]. Furthermore, user testing also highlighted the need for more direct language, particularly around problem-solving concepts [47]. The iterative study methodology used in this study enabled the participants to guide the development and provide their inputs at each stage to increase acceptability and feasibility.

#### Engagement

Although, overall, the apps were well received by young people, poor engagement (eg, not engaging at the recommended frequency or complete the full course of the intervention), measured through both self-report, intervention adherence, and data capture was a commonly reported issue. Many studies failed to achieve continued participation, with high rates of attrition [41,42,44]. In addition, app use often decreased markedly after the initial few weeks [41,42,44]. The results of some studies suggested that engagement (in the form of intervention completion) was related to the effectiveness the intervention [41,42]. Although engagement was problematic in many of the stand-alone interventions, engagement and study participation in a school setting seemed more promising [49]. For example, a proactive invitation for study participation in a school enabled 4 out of 5 eligible adolescents to participate in the “ready4life” life skills program [49]. This strategy consisted of individuals who were trained in the program to be delivered, giving arranged sessions lasting 30 minutes in participating vocational schools during regular school lessons reserved for health education. Within this session, the students were informed about and invited to participate in the study, including being informed about the study’s aims and assessments, reimbursement, and data protection.

#### Barriers to Engagement

Qualitative interviews and user feedback provided important insights about relevant barriers to engagement. The most frequently mentioned reasons for not using the app were that participants could not see the obvious benefits of using the app [41], lack of time or forgetting [42], and technical difficulties [41,50,51]. In a life skills training app for vocational students, participation was lower in male adolescents and among those reporting an immigrant background [49], although the reasons behind this poor engagement remained unclear.

#### What Do Young People Want From Apps?

There was some heterogeneity between studies in terms of the features and content that participants found acceptable and appropriate. For example, findings suggested that young people who experience homelessness tended to prefer both automated and self-help features compared with ones involving more direct human interaction [46]. However, participants in other studies valued both human interaction with professionals either via the app interface or through face-to-face contact and self-help features [47]. Human support was suggested as being helpful in offering both instruction and guidance as well as personalized support when needed. Numerous participants wanted opportunities to interact with peers [43,44,52] and even suggested connecting apps to social media [41]. Others also wanted the design of the apps to be more attractive (eg, improve the layout and create a more intuitive structure) and made suggestions about how gamifying apps could make them more interesting [41,43,47].

### Qualitative Study

#### Overview

We interviewed 13 young men in the United Kingdom (age: mean 18.7, SD 2.5 y), 2 in Spain (age: mean 17, SD 0 y), and 5 in Germany (age: mean 20.2, SD 1.6 y). In the United Kingdom, 62% (8/13) of the participants self-reported as ethnically White, compared with 50% (1/2) in Spain and 20% (1/5) in Germany (Table 3).

**Table 3:** Participant characteristics.

		United Kingdom (n=13)	Spain (n=2)	Germany (n=5)
Age (y), mean (SD)		18.7 (2.5)	17 (0)	20.2 (1.6)
**Ethnicity, n (%)**				
	Arab or Middle Eastern	0 (0)	0 (0)	4 (80)
	Asian	4 (31)	0 (0)	0 (0)
	White	8 (62)	1 (50)	1 (20)
	Other ethnic group	1 (8)	1 (50)	0 (0)
Refugee or an asylum seeker, n (%)		0 (0)	0 (0)	5 (100)
Chronic medical condition, n (%)		0 (0)	0 (0)	0 (0)
Disability, n (%)		1 (8)	0 (0)	0 (0)
**Educational attainment, n (%)**				
	Lower secondary school	6 (46)	2 (100)	4 (80)
	Upper secondary school	4 (31)	0 (0)	1 (20)
	Other higher education	1 (8)	0 (0)	0 (0)
	Undergraduate degree	1 (8)	0 (0)	0 (0)
	Postgraduate degree	1 (8)	0 (0)	0 (0)

In terms of participants’ mental health and well-being (Table 4), the mean Patient Health Questionnaire-9 score in the United Kingdom was 9.7 (SD 7.3) compared with 5 (SD 1.4) in Spain.

**Table 4:** Mental health and well-being.

Measures	United Kingdom (n=13), mean (SD)	Spain (n=2), mean (SD)	Germany (n=5), mean (SD)
WEMWBS^a^	44.2 (7.8)	51.5 (3.5)	—^b^
PHQ-9^c^	9.7 (7.3)	5 (1.4)	—
GAD-7^d^	6.5 (4.4)	8.5 (2.1)	—

^a^WEBWBS: Warwick-Edinburgh Mental Well-Being Scale.

^b^Not available.

^c^PHQ-9: Patient Health Questionnaire-9.

^d^GAD-7: Generalized Anxiety Disorder Assessment.

#### Acceptability and Feasibility

A key finding was that despite best efforts and financial incentives, recruiting underserved young male participants, especially in Spain and Germany, was challenging. This might suggest that these young people may not deem such an emotional competence app as relevant or useful to them, making recruitment and engagement problematic. We also assessed if the app was deemed acceptable (ie, useful, agreeable, palatable, or satisfactory) and appropriate (ie, relevant, suitable, or compatible). Overall, the app was viewed by participants in the United Kingdom, Spain, and Germany as being appropriate and relevant for young people of different ages and walks of life, as they thought that all young people had a smartphone and were adept at using technology:

So, I was able to learn about my feelings, I was able to evaluate how I actually felt today, concerning my feelings, if I was angry or I was sad. I was actually able to write them down in detail.

Participant in Germany

Several participants commented that the content of the app was best suited to university and school students. Another common view was that the app was better suited to those struggling with their mental health and that it was less relevant for those for whom things were going well. Many participants perceived the app to be aimed at improving mental health problems, as opposed to being a universal intervention intended to improve well-being, which represented a barrier to engagement. Of those who reported that the app was not relevant to them, they did see it as being of potential use to friends and family members who were stressed, anxious, or going through a difficult time:

There will be folks who maybe aren’t going through a good time in their lives, and they will need the app to feel... to understand themselves, mostly. And I think it’s relevant at any age, because I am lucky that I don’t think I need it as much as someone else who feels like that.

Participant in Spain

Partly it was important, partly it was not. I’ll give an example again, for example if a refugee came to Germany from a war zone, it’s going to be difficult, very difficult to find a topic that would fit him, for the future I mean, so the version now is already okay if you want all persons to use this app. Partly it’s already relevant and partly it’s not. If someone has mental problems or bad experiences, you cannot find such a topic in the app.

Participant in Germany

#### Engagement

Although some participants reported using the app regularly during the 4-week study period, a consistent finding was that participants tended to use the app most when they first downloaded it, with a marked reduction in use over time:

Uh, I probably used it about three times in the first week. And then not really that much at all I’m afraid.

Participant in the United Kingdom

I don’t know, I just dropped off using a little bit after a couple of weeks, but I’ve been trying to keep on top of doing that like the daily rating things and everything.... I kind of lost my motivation to use it.

Participant in the United Kingdom

#### Barriers to Engagement

We identified several barriers that hindered participants’ engagement and use of the app. These included the following: (1) repetitive and time-consuming app contents, (2) a paucity of new content and personalized or interactive tools (eg, matching mood to tools), (3) unclear instructions, (4) a lack of rationale for the app, (5) perceiving the app as not being relevant, (6) a lack of motivation, and (7) privacy concerns:

Yes, for example, I would not like to write in this diary, because I do not know if it would be one hundred percent anonymous and if others might read it. And maybe I have more privacy if I do not write it.

Participant in Germany

I think by now I would slowly stop using the app. It was nice up to this point, but I think for me I might need a step further now. To really deal with my personal problems and I don’t know how much an app like this can help and that rather an expert and therapy is needed.

Participant in Germany

For the asylum seekers and refugees in Germany, the language and content of the app was not suited to their needs. The participants would have preferred the app in their native language as some had to use translation programs to help access the content. Furthermore, specific topics of relevance to refugees were missing, such as dealing with asylum uncertainty, whereabouts of family members, and their living situation.

Finally, underserved young people, including asylum seekers and refugees, migrants, and those NEET, are more likely to experience financial deprivations and therefore less likely to pay directly for apps, especially for those that do not address their primary difficulties:

If it came to the point that I had to pay for it, I would look for free options.

Participant in Germany

## Discussion

### Summary of Findings

The use of mobile apps in mental health care continues to attract interest and investment; however, research geared toward understanding the needs of marginalized and underserved populations is still nascent. This study, focusing on the implementation of mental health apps in underserved young people, highlighted that little research exists to support the widespread adoption of these apps as a mental health intervention for marginalized and underserved groups. Findings from both our systematic review and qualitative study were largely consistent: markers of acceptability and usability were positive; however, engagement for underserved young people was low, which is notable given the widespread ownership of smartphones [55,56]. To date, research has focused primarily on efficacy studies rather than effectiveness and implementation in “real-world” settings and may have overestimated users’ “natural tendency” to adopt smartphone apps for their mental health and well-being [57]. Our findings suggest that despite the rapid proliferation of mobile mental health technology, the uptake and engagement of mental health apps among marginalized young people are low and remain a key implementation challenge.

Our data suggest that establishing and maintaining user commitment and engagement in the content of the intervention as intended is a pervasive challenge across mental health apps and marginalized populations, and premature dropout was prominent in nearly all the included studies. This is consistent with the literature that suggests that the majority of those offered these app-based interventions do not engage at the recommended frequency or complete the full course of treatment [58,59]. In this study, various app components were associated with engagement level, with the most engaging interventions providing young people with some form of associated real-human interaction and those having a more interactive interface. This aligned with other findings that the feedback of personalized information to participants is an especially important aspect of creating engaging and impactful digital tools [60]. Young people tend to quickly disengage if there are technical difficulties or if the app does not specifically target their perceived needs [41,50,51]. Furthermore, recruitment of marginalized groups to app-based studies is difficult. For instance, in this study, the use of advertisements, financial incentives, vouchers, and prize draw incentives seemed to be insufficient to recruit a significant number of participants in Spain and Germany.

Measuring engagement is a challenge that has likely contributed to our lack of knowledge on app components that effectively increase user engagement. Reporting engagement with mental health apps in intervention trials is highly variable, and a number of basic metrics of intervention engagement, such as rate of intervention uptake, weekly use patterns, and number of intervention completers, are available, yet not routinely reported [58,59]. The results of this study highlight the importance of objective engagement measures and that relying on positive subjective self-reports of usability, satisfaction, acceptability, and feasibility is insufficient to determine actual engagement. Furthermore, the findings suggest that apps involving human interactions with a professional (eg, therapist or counselor) or that are completed in a supervised setting tend to be more acceptable and effective and have higher engagement rates [47,48]. Our research suggests that similar to traditional face-to-face mental health services, app-based programs still face numerous barriers to reach marginalized youth, especially since the mental health apps available to the public do not seem to consider the unique developmental needs of these groups, participants do not seem to perceive an obvious benefit from using them, and some potential users prefer to interact with a professional face to face. Thus, it is also possible that the digital mental health field might be inadvertently contributing to mental health inequities among this population by not engaging marginalized groups sufficiently at the outset of research to ensure that the designed app meets their needs. However, for the studies included in this study that did engage these groups in the co-design of the apps, there was no notable improvement in engagement. Thus, we hope these findings encourage researchers and clinicians to think more critically of the role that mental health apps can truly have in addressing mental health equities among underserved groups.

As in other areas of mental health research, young people from LMICs were underrepresented in these studies, which typically originated from high-income settings, including the United States, Australia, and Canada. There are relatively few app-based interventions that were designed or adapted for young people in LMICs that have been rigorously evaluated or are even available in local languages [47,48]. Many living in LMIC regions, for example, adults in Asian countries, are often faced with apps that are not culturally relevant or in the right language [61]. These inequities are surprising given the high rates of smartphone use in Asia, even in rural regions [62]. Yet, it is still likely that youth in this region faced barriers related to data availability and more limited phone access, which will likely inhibit the broad implementation of apps beyond research studies [16]. Considerable work is required to ensure the availability of mental health apps that fit a wide range of user needs and preferences. It is important to ensure that the acceptability and feasibility of mental health apps for young people residing in LMICs are prioritized so that they are not further excluded from relevant mental health research.

Finally, a significant challenge is the lack of diversity in mental health app research participation, which limits our understanding of “real-world” efficacy and implementation for underserved and marginalized groups. While undoubtedly invaluable, and indeed deemed gold standard when evaluating efficacy of interventions, randomized controlled trial of mental health apps are not without flaws [63,64]. Trial recruitment is often highly selective due to stringent inclusion and exclusion criteria resulting in lower inclusion in research than one would expect from population estimates [65]. In the United Kingdom, the National Institute for Health and Care Research data have revealed that geographies with the highest burden of disease also have the lowest number of patients taking part in research [66]. The postcodes in which research recruitment is low also aligns closely to areas where earnings are the lowest and indexes of deprivation are the highest [66]. There are many reasons why some groups are underrepresented in research: language barriers, culturally inappropriate explanations, poor health literacy and the use of jargon, communication not being suitable for people with special learning needs, requirement to complete many administrative forms, negative financial impact in participating, lack of effective incentives for participation, or lack of clarity around incentives, and specific cultural and religious beliefs [66]. Failing to include a broad range of participants is problematic in that results may not be generalizable to a broad population.

### Limitations

Although this research was carefully executed and used a robust methodological approach with an exhaustive search strategy, it is not without limitations. Foremost, although the systematic review attempted to identify and include as many articles as possible, some papers may have been missed because of the inconsistencies in how feasibility and acceptability outcomes are recorded and reported. It was also difficult to ensure that all apps for this age group were identified because those aged between 15 and 25 years are harder to differentiate in adolescent and adult studies, meaning we might have missed some relevant studies where data could not be disaggregated by age. The exclusion of gray literature (eg, institutional reports and websites) may have also made us overlook potentially relevant apps, albeit lacking the quality assurance of peer-reviewed research. It is also likely that commercial organizations, including app companies, collect rich user demographic and engagement data but do not share it publicly, thus limiting our ability to conduct empirical analyses about the “real-world” acceptability, engagement, and implementation for specific populations. We did not analyze the extent to which publication bias may have influenced the results of our search, and, therefore, there may be a much higher number of mental health apps that have been developed with an underserved sample of young people, but due to their lack of efficacy or acceptability, these studies have not been submitted or accepted for publication. The sample sizes of many of the included studies were relatively low, which potentially limits their generalizability. However, we included all study designs so as to ensure that our learning from existing research was maximized. Furthermore, many of the studies included in the systematic review, as well as our qualitative study, had some form of language competency as an inclusion criterion (eg, English speaking), which likely excludes important perspectives from the results. For the qualitative study, we were only able to gather data from those who had used the app at least once and who were therefore somewhat engaged in the app. Despite our best efforts, we were unable to recruit participants who, following consent, had never then downloaded or used the app and so could not explore barriers to engagement for the least engaged young people or understand why the app was not appealing to those who chose not to proceed or take part. Those who did participate in this research were financially incentivized to do so and often highlighted the importance of this incentive in keeping them engaged. Therefore, we were unable to draw conclusions about the naturalistic engagement, feasibility, and acceptability of the app, if it were to be made available without payment in schools, universities, and health services or to be made commercially available on the app marketplace. It is also possible that social desirability bias (ie, a tendency to present reality to align with what is perceived to be socially acceptable) occurred during the interviews, whereby participants responded to the interview questions in a manner that they believed would be more acceptable to the study team, concealing their true opinions or experiences [67,68]. As previously noted by others, results may be subject to further bias in that findings could be led by more articulate young people, while it is more difficult to hear the voices of those who are less articulate or digitally literate [69]. Finally, it is also possible that the positionality of the research team, including our own experiences, backgrounds, and biases, impacted what information participants disclosed to the research team as well as the interpretation of the qualitative data in this study.

### Recommendations of Adaptations to Increase Acceptability, Feasibility, and Engagement

To overcome this complex engagement and implementation challenge, we have taken together our findings with relevant previous literature to generate 3 key suggestions about how to improve the feasibility and potential utility of apps for young people from marginalized and underserved populations.

### Increasing Participant Diversity in Mental Health Intervention Research

Studies should aim to prioritize the inclusion of marginalized groups in trials testing the effectiveness of digital interventions by intentionally planning recruitment efforts aimed to reach these communities [70]. First, steps can be taken to build trust, connections, and credibility between the research team and these communities. NHS England [66] suggests involving representatives from those groups during the inception and implementation of recruitment efforts. This approach ensures that the intervention is relevant to the target group by meeting their preferences and needs, incorporating culturally salient factors relevant for recruitment efforts, addressing concerns about community mistrust and participant resource constraints, and establishing partnerships with key community stakeholders that can be gatekeepers in the community [14,71]. These strategies are likely to improve research accessibility, recruitment, and retention. Research teams need to ensure that the findings and any actionable takeaways from the research conducted with the participants are shared with them by asking participants how they would like to receive this information (eg, verbal, written, or via a trusted advocate). Equally important is to explain that the research process can be slow. These steps help create a positive legacy for the research project and build trust between individuals and public institutions, helping future health researchers to further address underrepresentation of marginalized groups in digital research.

### Identifying and Addressing Needs and Preferences of Underserved and Marginalized Groups by Using Human-Centered Design Principles

A comprehensive understanding of the needs, challenges, and life circumstances of the target population is a key implementation driver for designing relevant, engaging, and effective mental health apps. This knowledge is particularly important when the app is a stand-alone intervention received during daily life outside of traditional psychotherapy or human support [50]. This goal can be best achieved through a participatory approach, which reflects a growing recognition among intervention researchers and developers that end users need to be involved in the creation of interventions and their future iterations [47,72]. This process may involve a series of stages, including (1) person-centered co-design to ensure that tools are developed to be acceptable to the underserved or marginalized populations as well as meet their specific needs, life circumstances, and cultural norms [47]; (2) iterative testing that incorporates users’ feedback on a rolling basis to ensure the relevance of the intervention [43,47,72]; and (3) changes and adaptations needed to meet users’ needs in “real-world” settings including consideration of economic viability and implementation [27].

Especially relevant for the underserved and marginalized groups is the need (or lack thereof) to culturally adapt app interventions for specific racial, ethnic, or cultural groups through this person-centered design. In traditional face-to-face interventions, some have argued that all treatments need to be culturally adapted to ensure their validity, relevance, and effectiveness since these interventions are often developed with individuals who can be substantially different from some marginalized populations [73]. Similar to culturally adapted face-to-face interventions [74-76], culturally adapted digital mental health interventions seem to be effective [77,78]. However, there is no evidence that these culturally adapted interventions outperform the original programs [79,80]. Given that culturally adapting digital interventions is a time-consuming and resource-intensive process, this approach may not be sustainable and limit the dissemination and implementation impact of app programs [28]. In lieu of culturally adapting digital interventions without careful consideration, Ramos and Chavira [28] recommend using information gathered through person-centered approaches to integrate culture into the use of already available digital interventions (including apps), using an idiographic, flexible, and personalized approach. This strategy may have a broader implementation and dissemination potential, given that few researchers and clinicians are in a position to develop new apps.

### Embedding Apps Within Existing Care Structures

Several systematic reviews and meta-analyses have demonstrated that app-based mental health interventions with a human-support component are more effective and more acceptable than stand-alone, fully automatized, or self-administered apps [13,25,81]. Young people seem to want practical skills and usable tools to apply to their current daily life stressors to improve their well-being and functioning. Intervention engagement is enhanced if the intervention serves an obvious purpose, is relevant, and has a clear rationale and instructions, and embedding these interventions within the systems and structures that are already working with users (eg, clinical services, schools, universities, and community agencies) will likely improve implementation. Considering the broad and highly varied nature of intervention formats and modalities, it may be useful for future research to focus on identifying core components of app-based interventions (ie, active ingredients of interventions associated with uptake, adherence, and clinical outcomes) that will allow such integration of app interventions into the varied context of care for marginalized youth.

### Conclusions

Despite the enthusiasm that has surrounded the potential of digital technologies to revolutionize mental health and health care service delivery, little evidence yet supports the use of mental health apps for marginalized and underserved young people. Despite the substantial financial and human investment directed to the development of mental health apps over several years, only a small proportion have empirical evidence to support their effectiveness, and there have been few attempts to develop or adapt interventions to meet some of the more unique and heterogeneous needs of diverse groups of young people. Although acceptability seems to be good, engagement is poor and attrition is high, particularly if not supported by in-person elements. Given that most interventions are implemented in high-income countries, very little is known about the generalizability of the findings to LMICs and to a range of adolescents and young people with different socioeconomic, cultural, and racial backgrounds. In this paper, we have drawn several insights about the feasibility and acceptability of mental health apps for underserved young people that may be useful to future app-based mental health promotion and treatment projects. However, before the widespread adoption and scaling-up of digital mental health interventions progresses further, especially for more vulnerable and underserved populations and in settings with limited resources, a greater understanding is needed on the unique barriers faced by these groups in accessing treatment and the types of services young people themselves prefer (eg, standard vs digital) followed by more rigorous and consistent demonstrations of feasibility, effectiveness, and cost-effectiveness.

## Appendix

Multimedia Appendix 1 Search strategy.

Multimedia Appendix 2 Topic guide.

Multimedia Appendix 3 Study quality assessment.

## Abbreviations

ECoWeB: Emotional Competence for Well-Being
LMIC: low- and middle-income country
MMAT: Mixed Methods Appraisal Tool
NEET: not in education, employment, or training
PRISMA: Preferred Reporting Items for Systematic Reviews and Meta-Analyses

## Data Availability

The data extracted to support the findings of the systematic review are available from the corresponding author upon reasonable request. Due to the confidential and sensitive nature of the interview transcripts, qualitative data will not be made available.
