# Supplementary material for: The Acceptability, Engagement, and Feasibility of Mental Health Apps for Marginalized and Underserved Young People: Systematic Review and Qualitative Study
Source: J Med Internet Res. 2024 Jul 30;26:e48964. doi: 10.2196/48964 (PMC11322694; doi:10.2196/48964)
Supplement: Multimedia Appendix 1 [file jmir_v26i1e48964_app1.docx]

Search made on 22.06.21 in Embase (Ovid), Medline (Ovid), PsycInfo (Ovid), Cochrane Central and Cochrane Database.

Searched key words related to:

1) underserved populations:

- marginali*, disadvantage*, vulnerable, underrepresented, underserved, at-risk.
- not in education, employment or training (NEET);
- out-of-school, school dropout;
- Apprentic*;
- teenage parent*;
- ethnic minorit*;
- sexual minorit*, LGBT*
- homeless;
- socioeconomically deprived; low-income; poor; low SES
- Substance, drug, alcohol, addict*
- under State/statutory care, foster care;
- refugee; asylum
- Migrant*; immigrant*
- disabled; disability
- Rural, remote;
- Crim*, Justice, incarcerated, delinquent

2) adolescents and young people:

3) mental health

4) mobile apps;

5) acceptability and feasibility implementation outcomes

Results of the search:

| Ovid Embase | 5916 |
| --- | --- |
| Ovid Medline | 2809 |
| Ovid PsycINFO | 665 |
| Cochrane CENTRAL | 4800 |
| Cochrane Database | 138 |
| Total | 14,328 |
| Total after deduplication | 11,539 |

**Limits: Jan 2008 to Dec 2021 (or current), title and abstract.**

**Embase**

1 (social exclusion or [vulnerable population](https://ezproxy-prd.bodleian.ox.ac.uk:2483/ovid-b/ovidweb.cgi?&Controlled+Vocabulary=Mapping%7c8&Return=mapping&S=BIBEFPDPFPEBMCFNIPPJEGOGGBGLAA00) or [risk](https://ezproxy-prd.bodleian.ox.ac.uk:2483/ovid-b/ovidweb.cgi?&Controlled+Vocabulary=Mapping%7c6&Return=mapping&S=BIBEFPDPFPEBMCFNIPPJEGOGGBGLAA00) or [medically underserved](https://ezproxy-prd.bodleian.ox.ac.uk:2483/ovid-b/ovidweb.cgi?&Controlled+Vocabulary=Mapping%7c0&Return=mapping&S=BIBEFPDPFPEBMCFNIPPJEGOGGBGLAA00) or [minority group](https://ezproxy-prd.bodleian.ox.ac.uk:2483/ovid-b/ovidweb.cgi?&Controlled+Vocabulary=Mapping%7c1&Return=mapping&S=BIBEFPDPFPEBMCFNIPPJEGOGGBGLAA00) or NEET status or [school dropout](https://ezproxy-prd.bodleian.ox.ac.uk:2483/ovid-b/ovidweb.cgi?&Controlled+Vocabulary=Mapping%7c0&Return=mapping&S=BIBEFPDPFPEBMCFNIPPJEGOGGBGLAA00) or abstenteeism or adolescent parent or [ethnic group](https://ezproxy-prd.bodleian.ox.ac.uk:2483/ovid-b/ovidweb.cgi?&Controlled+Vocabulary=Mapping%7c0&Return=mapping&S=DOEHFPDKJMEBMCDCJPPJFFBFMNELAA00) or [homeless youth](https://ezproxy-prd.bodleian.ox.ac.uk:2483/ovid-b/ovidweb.cgi?&Controlled+Vocabulary=Mapping%7c1&Return=mapping&S=DOEHFPDKJMEBMCDCJPPJFFBFMNELAA00) or homeless person or ["sexual and gender minority"](https://ezproxy-prd.bodleian.ox.ac.uk:2483/ovid-b/ovidweb.cgi?&Controlled+Vocabulary=Mapping%7c0&Return=mapping&S=DOEHFPDKJMEBMCDCJPPJFFBFMNELAA00) or [LGBTQIA+ people](https://ezproxy-prd.bodleian.ox.ac.uk:2483/ovid-b/ovidweb.cgi?&Controlled+Vocabulary=Mapping%7c7&Return=mapping&S=DOEHFPDKJMEBMCDCJPPJFFBFMNELAA00) or [socioeconomics](https://ezproxy-prd.bodleian.ox.ac.uk:2483/ovid-b/ovidweb.cgi?&Controlled+Vocabulary=Mapping%7c5&Return=mapping&S=BIBEFPDPFPEBMCFNIPPJEGOGGBGLAA00) or [social class](https://ezproxy-prd.bodleian.ox.ac.uk:2483/ovid-b/ovidweb.cgi?&Controlled+Vocabulary=Mapping%7c2&Return=mapping&S=BIBEFPDPFPEBMCFNIPPJEGOGGBGLAA00) or [poverty](https://ezproxy-prd.bodleian.ox.ac.uk:2483/ovid-b/ovidweb.cgi?&Controlled+Vocabulary=Mapping%7c7&Return=mapping&S=DOEHFPDKJMEBMCDCJPPJFFBFMNELAA00) or social status or [substance abuse](https://ezproxy-prd.bodleian.ox.ac.uk:2483/ovid-b/ovidweb.cgi?&Controlled+Vocabulary=Mapping%7c0&Return=mapping&S=DOEHFPDKJMEBMCDCJPPJFFBFMNELAA00) or [addiction](https://ezproxy-prd.bodleian.ox.ac.uk:2483/ovid-b/ovidweb.cgi?&Controlled+Vocabulary=Mapping%7c0&Return=mapping&S=DOEHFPDKJMEBMCDCJPPJFFBFMNELAA00) or [cannabis addiction](https://ezproxy-prd.bodleian.ox.ac.uk:2483/ovid-b/ovidweb.cgi?&Controlled+Vocabulary=Mapping%7c3&Return=mapping&S=DOEHFPDKJMEBMCDCJPPJFFBFMNELAA00) or alcohol abuse or foster care or refugee or [migrant](https://ezproxy-prd.bodleian.ox.ac.uk:2483/ovid-b/ovidweb.cgi?S=DOEHFPDKJMEBMCDCJPPJFFBFMNELAA00&Controlled+Vocabulary=thes+migrant&) or [disabled person](https://ezproxy-prd.bodleian.ox.ac.uk:2483/ovid-b/ovidweb.cgi?&Controlled+Vocabulary=Mapping%7c0&Return=mapping&S=DOEHFPDKJMEBMCDCJPPJFFBFMNELAA00) or [disability](https://ezproxy-prd.bodleian.ox.ac.uk:2483/ovid-b/ovidweb.cgi?&Controlled+Vocabulary=Mapping%7c1&Return=mapping&S=DOEHFPDKJMEBMCDCJPPJFFBFMNELAA00) or [language disability](https://ezproxy-prd.bodleian.ox.ac.uk:2483/ovid-b/ovidweb.cgi?&Controlled+Vocabulary=Mapping%7c9&Return=mapping&S=DOEHFPDKJMEBMCDCJPPJFFBFMNELAA00) or physical disability or [rural population](https://ezproxy-prd.bodleian.ox.ac.uk:2483/ovid-b/ovidweb.cgi?&Controlled+Vocabulary=Mapping%7c2&Return=mapping&S=DOEHFPDKJMEBMCDCJPPJFFBFMNELAA00) or [juvenile delinquency](https://ezproxy-prd.bodleian.ox.ac.uk:2483/ovid-b/ovidweb.cgi?&Controlled+Vocabulary=Mapping%7c0&Return=mapping&S=DOEHFPDKJMEBMCDCJPPJFFBFMNELAA00) or criminal behavior or offender or criminal justice or juvenile).ti,ab.

Hits: 2862747

2 (minor* or boy* or girl* or schoolchild* or pupil* or adolescen* or juvenil* or youth* or teen* or pubescen* or prepub* or pre-pub* or preadolescen* or pre-adolescen* or "young adult*" or "young people" or "young person" or "young man" or "young men" or "young woman" or "young women" or student*).ti,ab.

Hits: 1151195

3 ("mental difficult*" or "mental health challenge*" or "mental health difficult*" or "mental health problem*" or "mental challenge*" or "mental well$being" or "mental health well$being" or "mental illness" or "mental health illness" or "mood disorder*" or "mental health" or "attention deficit disorder" or ADHD or "behavio$r disorder*" or "communication disorder*" or "conduct disorder*" or "emotional adjustment*" or "emotional* disturb*" or "intellectual development disorder*" or "learning disorder*" or "psychiatric patient*" or psychopatholog* or suicid* or "thought disturbance*" or psychotherap* or "child psychopatholog*" or psychiatr* or anxiety or depression or suicid* or stress or psychos* or psychotic* or bipolar* or schizophren* or schizoaff* or "eating disorder*" or bulimi* or anorexi*).ti,ab.

Hits: 1593189

4 ("cell* phone*" or "mobile phone*" or smartphone* or "smart phone*" or "personal digital assistant*" or PDA or internet* or app or apps or application* or "mobile device*" or "mobile app*" or smartphone* or "mobile phone*" or digital* or technolog* or computer* or mhealth* or mhealth or mobile health or ehealth or "electronic health" or "social media" or phone or online or web-based).ti,ab.

Hits: 2037812

5 (treatment* or intervention* or therap* or training or support* or help* or assistan* or development or evaluation or trial* or RCT* or evidence or efficacy or effectiveness or implementation or case-control or fidelity or assessment or clinical stud* or testing or acceptability or feasibility or adoption or clinical trial or randomized controlled trial).ti,ab.

Hits: 11093149

6) 1 and 2 and 3 and 4 and 5 and 6.

Hits: 5916

**Medline**

1 ([Vulnerable Populations](https://ezproxy-prd.bodleian.ox.ac.uk:2483/ovid-b/ovidweb.cgi?&Controlled+Vocabulary=Mapping%7c0&Return=mapping&S=OALHFPLIICEBLCDIJPPJHGHGMFDJAA00) or Medically Underserved Area or risk or [Unemployment](https://ezproxy-prd.bodleian.ox.ac.uk:2483/ovid-b/ovidweb.cgi?&Controlled+Vocabulary=Mapping%7c8&Return=mapping&S=OALHFPLIICEBLCDIJPPJHGHGMFDJAA00) or [Student Dropouts](https://ezproxy-prd.bodleian.ox.ac.uk:2483/ovid-b/ovidweb.cgi?&Controlled+Vocabulary=Mapping%7c0&Return=mapping&S=IKPBFPKGAJEBGCNLIPPJKHPEJIJDAA00) or Pregnancy in Adolescence Minority Groups or Ethnic Groups or [African Americans](https://ezproxy-prd.bodleian.ox.ac.uk:2483/ovid-b/ovidweb.cgi?&Controlled+Vocabulary=Mapping%7c6&Return=mapping&S=IKPBFPKGAJEBGCNLIPPJKHPEJIJDAA00) or Homeless Youth or Homeless Persons or "Sexual and Gender Minorities" or Bisexuality or Homosexuality, Male or Homosexuality, Female or Transgender Persons or Homosexuality or Transsexualism or Psychosocial Deprivation or [Socioeconomic Factors](https://ezproxy-prd.bodleian.ox.ac.uk:2483/ovid-b/ovidweb.cgi?&Controlled+Vocabulary=Mapping%7c0&Return=mapping&S=IKPBFPKGAJEBGCNLIPPJKHPEJIJDAA00) or [Social Class](https://ezproxy-prd.bodleian.ox.ac.uk:2483/ovid-b/ovidweb.cgi?&Controlled+Vocabulary=Mapping%7c5&Return=mapping&S=IKPBFPKGAJEBGCNLIPPJKHPEJIJDAA00) or Poverty or Substance-Related Disorders or [Alcoholism](https://ezproxy-prd.bodleian.ox.ac.uk:2483/ovid-b/ovidweb.cgi?&Controlled+Vocabulary=Mapping%7c0&Return=mapping&S=IKPBFPKGAJEBGCNLIPPJKHPEJIJDAA00) or [Foster Home Care](https://ezproxy-prd.bodleian.ox.ac.uk:2483/ovid-b/ovidweb.cgi?&Controlled+Vocabulary=Mapping%7c0&Return=mapping&S=IKPBFPKGAJEBGCNLIPPJKHPEJIJDAA00) or Refugees or ["Emigration and Immigration"](https://ezproxy-prd.bodleian.ox.ac.uk:2483/ovid-b/ovidweb.cgi?&Controlled+Vocabulary=Mapping%7c8&Return=mapping&S=IKPBFPKGAJEBGCNLIPPJKHPEJIJDAA00) or Intellectual Disability or [Learning Disabilities](https://ezproxy-prd.bodleian.ox.ac.uk:2483/ovid-b/ovidweb.cgi?&Controlled+Vocabulary=Mapping%7c0&Return=mapping&S=IKPBFPKGAJEBGCNLIPPJKHPEJIJDAA00) or disabled children or [Rural Population](https://ezproxy-prd.bodleian.ox.ac.uk:2483/ovid-b/ovidweb.cgi?&Controlled+Vocabulary=Mapping%7c0&Return=mapping&S=IKPBFPKGAJEBGCNLIPPJKHPEJIJDAA00) or Juvenile Delinquency or Prisoners or Crime or Criminals or [Prisons](https://ezproxy-prd.bodleian.ox.ac.uk:2483/ovid-b/ovidweb.cgi?&Controlled+Vocabulary=Mapping%7c8&Return=mapping&S=IKPBFPKGAJEBGCNLIPPJKHPEJIJDAA00)).ti,ab.

Hits: 1686573

2 (minor* or boy* or girl* or schoolchild* or pupil* or adolescen* or juvenil* or youth* or teen* or pubescen* or prepub* or pre-pub* or preadolescen* or pre-adolescen* or young adult* or young people or young person or young man or young men or young woman or young women or student*).ti,ab.

Hits: 782554

3 (mental health or mental disorders or mental difficult* or mental health problem or mental well$being or mental disease or mental health services or mentally ill persons or mental challenge or mental health challenge* or mental health difficult* or mental health well$being or mental illness or mental health illness or emotional competence or emotional adjustment or emotional disturb* or emotional difficult* or well-being or well being or wellbeing or psychological adjustment or adolescent psychotherapy or adolescent psychiatry or psychopathology or preventive mental health or abnormal psychology or mood disorder or anxiety or depression or stress or psychos* or eating disorder*" or bulimi* or anorexi* or affective symptoms or affective disorder* or suicid* or psychos* or psychotic* or bipolar* or schizophren* or schizoaff* or self-harm or self-injur* or thought disturb* or child psychiatry or attention deficit disorder or adhd or behavio$r disorder* or communication disorder* or conduct disorder* or intellectual development disorder* or psychiatric patient* or psychotherap* or child psychopatholog*).ti,ab.

Hits: 1133218

4) (Cell Phone or handheld or hand held or cell* phone* or mobile phone* or smartphone* or smart phone* or personal digital assistant* or PDA or Mobile Applications or device* or internet* or app or apps or application* or mobile device* or mobile app* or digital* or technolog* or mhealth* or mobile health or ehealth or electronic health or phone or online or web-based).ti,ab.

Hits: 1695125

5) (treatment* or intervention* or therap* or training or support* or help* or assistan* or Clinical Trials or development or evaluation or trial* or RCT* or evidence or efficacy or effectiveness or implementation or case-control or fidelity or assessment or "clinical stud*" or testing or acceptability or feasibility or adoption or [Patient Participation](https://ezproxy-prd.bodleian.ox.ac.uk:2483/ovid-b/ovidweb.cgi?&Controlled+Vocabulary=Mapping%7c9&Return=mapping&S=IKPBFPKGAJEBGCNLIPPJKHPEJIJDAA00) or [Pilots](https://ezproxy-prd.bodleian.ox.ac.uk:2483/ovid-b/ovidweb.cgi?&Controlled+Vocabulary=Mapping%7c0&Return=mapping&S=IKPBFPKGAJEBGCNLIPPJKHPEJIJDAA00) or Program Development or Program Evaluation or Primary prevention or secondary prevention or Health Promotion or "Outcome and Process Assessment, Health Care" or Outcome Assessment, Health Care or Patient Outcome Assessment or Patient Reported Outcome Measures or Treatment Outcome).ti,ab.

Hits: 7641219

6) 1 and 2 and 3 and 4 and 5.

Hits: 2792

PsycInfo

1) (Disadvantaged or [Minority Groups](https://ezproxy-prd.bodleian.ox.ac.uk:2483/ovid-b/ovidweb.cgi?&Controlled+Vocabulary=Mapping%7c8&Return=mapping&S=OOHIFPBANCEBOCFLIPPJLHEHFILLAA00) or [Blacks](https://ezproxy-prd.bodleian.ox.ac.uk:2483/ovid-b/ovidweb.cgi?&Controlled+Vocabulary=Mapping%7c9&Return=mapping&S=OOHIFPBANCEBOCFLIPPJLHEHFILLAA00) or [At Risk Populations](https://ezproxy-prd.bodleian.ox.ac.uk:2483/ovid-b/ovidweb.cgi?&Controlled+Vocabulary=Mapping%7c11&Return=mapping&S=OOHIFPBANCEBOCFLIPPJLHEHFILLAA00) or ["Racial and Ethnic Groups"](https://ezproxy-prd.bodleian.ox.ac.uk:2483/ovid-b/ovidweb.cgi?&Controlled+Vocabulary=Mapping%7c10&Return=mapping&S=OOHIFPBANCEBOCFLIPPJLHEHFILLAA00) or Marginalization or [Social Exclusion](https://ezproxy-prd.bodleian.ox.ac.uk:2483/ovid-b/ovidweb.cgi?&Controlled+Vocabulary=Mapping%7c10&Return=mapping&S=OOHIFPBANCEBOCFLIPPJLHEHFILLAA00) or Unemployment or School to Work Transition or School Dropouts or Adolescent mothers OR adolescent pregnancy or adolescent fathers or Sexual Minority Groups or [LGBTQ](https://ezproxy-prd.bodleian.ox.ac.uk:2483/ovid-b/ovidweb.cgi?&Controlled+Vocabulary=Mapping%7c7&Return=mapping&S=BGPJFPHGGBEBGCAHIPPJLFEHGAAEAA00) or Deprivation or [Socioeconomic Status](https://ezproxy-prd.bodleian.ox.ac.uk:2483/ovid-b/ovidweb.cgi?&Controlled+Vocabulary=Mapping%7c0&Return=mapping&S=OOHIFPBANCEBOCFLIPPJLHEHFILLAA00) or Social Deprivation or Poverty or Drug Abuse or Foster Care or [Foster Children](https://ezproxy-prd.bodleian.ox.ac.uk:2483/ovid-b/ovidweb.cgi?&Controlled+Vocabulary=Mapping%7c13&Return=mapping&S=OOHIFPBANCEBOCFLIPPJLHEHFILLAA00) or [Refugees](https://ezproxy-prd.bodleian.ox.ac.uk:2483/ovid-b/ovidweb.cgi?&Controlled+Vocabulary=Mapping%7c0&Return=mapping&S=OOHIFPBANCEBOCFLIPPJLHEHFILLAA00) or Immigration or Developmental Disabilities or Disabilities or Learning Disabilities or Multiple Disabilities or Reading Disabilities or Rural Environments or Juvenile Delinquency or Criminal Behavior or Crime or Incarceration or Prisoners or Prisons or Correctional Institutions or Criminal Offenders or Criminal Rehabilitation).ti,ab.

Hits: 147481

2) (minor* or boy* or girl* or schoolchild* or pupil* or adolescen* or juvenil* or youth* or teen* or pubescen* or prepub* or pre-pub* or preadolescen* or pre-adolescen* or "young adult*" or "young people" or "young person" or "young man" or "young men" or "young woman" or "young women" or student* or adolescent psychiatry or adolescent psychotherapy or students).ti,ab.

Hits: 525116

3) (mental health or "mental difficult*" or Mental disorders or "mental health challenge*" or "mental health difficult*" or "mental health problem*" or "mental challenge*" or "mental well$being" or "mental health well$being" or "mental illness" or "mental health illness" or "mood disorder*" or "attention deficit disorder" or ADHD or "behavio$r disorder*" or "communication disorder*" or "conduct disorder*" or "emotional adjustment*" or "emotional* disturb*" or "intellectual development disorder*" or "learning disorder*" or "psychiatric patient*" or psychopatholog* or suicid* or "thought disturbance*" or psychotherap* or "child psychopatholog*" or psychiatr* or anxiety or depression or suicid* or stress or psychos* or psychotic* or bipolar* or schizophren* or schizoaff* or "eating disorder*" or bulimi* or anorexi*).ti,ab.

Hits: 595144

4) (cell* phone* or Mobile applications or mobile app* or Mobile device* or Mobile phone* or handheld or hand held or smartphone* or smart phone* or Mobile Health or Digital Interventions or personal digital assistant* or PDA or Internet* or web-based or online or technolog* or app or apps or application* or digital* or technolog* or mhealth* or mhealth or ehealth or electronic health or social media or phone).ti,ab.

Hits: 293857

5) (Evidence Based Practice or Intervention or Treatment or Treatment Effectiveness Evaluation or Treatment Outcomes or Adaptation or [Psychological Engagement](https://ezproxy-prd.bodleian.ox.ac.uk:2483/ovid-b/ovidweb.cgi?&Controlled+Vocabulary=Mapping%7c2&Return=mapping&S=OOHIFPBANCEBOCFLIPPJLHEHFILLAA00) or [Clinical Trials](https://ezproxy-prd.bodleian.ox.ac.uk:2483/ovid-b/ovidweb.cgi?&Controlled+Vocabulary=Mapping%7c11&Return=mapping&S=OOHIFPBANCEBOCFLIPPJLHEHFILLAA00) or Clinical Psychology or Health promotion or [Prevention](https://ezproxy-prd.bodleian.ox.ac.uk:2483/ovid-b/ovidweb.cgi?&Controlled+Vocabulary=Mapping%7c4&Return=mapping&S=OOHIFPBANCEBOCFLIPPJLHEHFILLAA00) or development or evaluation or trial* or RCT* or evidence or efficacy or effectiveness or implementation or case-control or fidelity or assessment or "clinical stud*" or testing or acceptability or feasibility or adoption).ti,ab.

Hits: 1267824

6) 1 and 2 and 3 and 4 and 5.

Hits: 665

**Limits: 2008 to present**

**Results: 1463**

**Cochrane Database of Systematic Reviews**

Issue 6 of 12, June 2021

Limits: Jan 2008 to July 2021. Title, abstract and keywords. Results: 138

**Cochrane Central Register of Controlled Trials**

Issue 6 of 12, June 2021

Limits: Jan 2008 to July 2021. Title, abstract and keywords. Results: 4800

1 (Marginalization, Social or Disadvantaged or Disadvantagement, Cultural or Vulnerable Populations or Underserved Area, Medically or Underserved Populations or Underserved patients or Dropout*, School or Dropout*, Student or Teenage Pregnanc* or ethnic group* or Homeless Person* or Homeless Youth* or Homelessness or Minorities, Gender or Minorities, Sexual or Minority Group* or Minority Health or LGBT Person* or Deprivation, Psychosocial or Socioeconomic Factors or Socioeconomic Status or Social Class* or Low-Income Population* or Poor, Working or Poverty or Substance Abuse or Addiction, Alcohol or Addiction, Drug or Foster Youth* or Foster Home Care or Refugee* or Refugee*, Political or Asylum Seeker* or Asylum Seeker, Political or Migrant* or Immigrant* or Immigrant*, Unauthorized or Immigrant*, Undocumented or Disabilities, Acquired Reading or Disabilities, Adult Learning or Disabilities, Communication or Disabilities, Developmental or Disabilities, Developmental Academic or Disabilities, Developmental Reading or Disabilities, Intellectual or Disabilities, Learning or Disabilities, People with or Disabilities, Persons with or Rural communit* or Crime or criminal or Delinquency, Juvenile or Criminal Behavior* or Criminal justice or Prison*).

2 (minor* or boy* or girl* or schoolchild* or pupil* or adolescen* or juvenil* or youth* or teen* or pubescen* or prepub* or pre-pub* or preadolescen* or pre-adolescen* or "young adult*" or "young people" or "young person" or "young man" or "young men" or "young woman" or "young women" or student*)

3 ("mental difficult*" or "mental health challenge*" or "mental health difficult*" or "mental health problem*" or "mental challenge*" or "mental well$being" or "mental health well$being" or "mental illness" or "mental health illness" or "mood disorder*" or "mental health" or "attention deficit disorder" or ADHD or "behavio$r disorder*" or "communication disorder*" or "conduct disorder*" or "emotional adjustment*" or "emotional* disturb*" or "intellectual development disorder*" or "learning disorder*" or "psychiatric patient*" or psychopatholog* or suicid* or "thought disturbance*" or psychotherap* or "child psychopatholog*" or psychiatr* or anxiety or depression or suicid* or stress or psychos* or psychotic* or bipolar* or schizophren* or schizoaff* or "eating disorder*" or bulimi* or anorexi*)

4 (internet* or app or apps or application* or "mobile device*" or "mobile app*" or smartphone* or "mobile phone*" or digital* or technolog* or computer* or mhealth* or mobile health or ehealth or "electronic health" or "social media" or phone or online or web-based)

5 (Acceptability of Health$care or feasibility stud* or pilot stud* or Clinical Trial or Evaluation Research or treatment* or intervention* or therap* or training or support* or help* or assistan* or Implementation Science* or Evidence Based Medicine or Evidence Based Healthcare or Impact Assessment*, Health or Assessment, Health Impact or Program Accessibility or Program Appropriateness or Program Development or Program Effectiveness or Program Efficienc* or Program Evaluation or Preventative Care or Preventative Medicine or Promotion, Health or Outcome* Assessment, Health Care)

**Syntax:**

(Marginalization, Social or Disadvantaged or Disadvantagement, Cultural or Vulnerable Populations or Underserved Area, Medically or Underserved Populations or Underserved patients or Dropout*, School or Dropout*, Student or Teenage Pregnanc* or ethnic group* or Homeless Person* or Homeless Youth* or Homelessness or Minorities, Gender or Minorities, Sexual or Minority Group* or Minority Health or LGBT Person* or Deprivation, Psychosocial or Socioeconomic Factors or Socioeconomic Status or Social Class* or Low-Income Population* or Poor, Working or Poverty or Substance Abuse or Addiction, Alcohol or Addiction, Drug or Foster Youth* or Foster Home Care or Refugee* or Refugee*, Political or Asylum Seeker* or Asylum Seeker, Political or Migrant* or Immigrant* or Immigrant*, Unauthorized or Immigrant*, Undocumented or Disabilities, Acquired Reading or Disabilities, Adult Learning or Disabilities, Communication or Disabilities, Developmental or Disabilities, Developmental Academic or Disabilities, Developmental Reading or Disabilities, Intellectual or Disabilities, Learning or Disabilities, People with or Disabilities, Persons with or Rural communit* or Crime or criminal or Delinquency, Juvenile or Criminal Behavior* or Criminal justice or Prison*) AND (minor* or boy* or girl* or schoolchild* or pupil* or adolescen* or juvenil* or youth* or teen* or pubescen* or prepub* or pre-pub* or preadolescen* or pre-adolescen* or "young adult*" or "young people" or "young person" or "young man" or "young men" or "young woman" or "young women" or student*) AND ("mental difficult*" or "mental health challenge*" or "mental health difficult*" or "mental health problem*" or "mental challenge*" or "mental well$being" or "mental health well$being" or "mental illness" or "mental health illness" or "mood disorder*"or "mental health" or "attention deficit disorder" or ADHD or "behavio$r disorder*" or "communication disorder*" or "conduct disorder*" or "emotional adjustment*" or "emotional* disturb*" or "intellectual development disorder*" or "learning disorder*" or "psychiatric patient*" or psychopatholog* or suicid* or "thought disturbance*" or psychotherap* or "child psychopatholog*" or psychiatr* or anxiety or depression or suicid* or stress or psychos* or psychotic* or bipolar* or schizophren* or schizoaff* or "eating disorder*" or bulemi* or anorexi*) AND (internet* or app or apps or application* or "mobile device*" or "mobile app*" or smartphone* or "mobile phone*" or digital* or technolog* or computer* or mhealth* or mobile health or ehealth or "electronic health" or "social media" or phone or online or web-based) AND (Acceptability of Health$care or feasibility stud* or pilot stud* or Clinical Trial or Evaluation Research or treatment* or intervention* or therap* or training or support* or help* or assistan* or Implementation Science* or Evidence Based Medicine or Evidence Based Healthcare or Impact Assessment*, Health or Assessment, Health Impact or Program Accessibility or Program Appropriateness or Program Development or Program Effectiveness or Program Efficienc* or Program Evaluation or Preventative Care or Preventative Medicine or Promotion, Health or Outcome* Assessment, Health Care)

Screened the title of 11539 entries. Excluded: 10061, Included: 1478. Finished on 22/09/2021.

Second stage: abstract screening of 1478 titles. Finished on 18/10/2021

| Undecided | 0 |
| --- | --- |
| Maybe | 378 |
| Included | 122 |
| Excluded | 978 |
| Conflict | 0 |

**After full-text screening the “maybes”, 176 texts were included**

**Updated search on 28^th^ September 2023**

3 resources selected:

Embase 1974 to present

Medline (Ovid MEDLINE® Epub Ahead of Print, In-Process & Other Non-Indexed Citations, Ovid MEDLINE® Daily and Ovid MEDLINE®) 1946 to present

PsycINFO 1806 to present

1 (social exclusion or vulnerable population or risk or medically underserved or minority group or NEET status or school dropout or abstenteeism or adolescent parent or ethnic group or homeless youth or homeless person or "sexual and gender minority" or LGBTQIA+ people or socioeconomics or social class or poverty or social status or substance abuse or addiction or cannabis addiction or alcohol abuse or foster care or refugee or migrant or disabled person or disability or language disability or physical disability or rural population or juvenile delinquency or criminal behavior or offender or criminal justice or juvenile).ti,ab. 8348067

2 (minor* or boy* or girl* or schoolchild* or pupil* or adolescen* or juvenil* or youth* or teen* or pubescen* or prepub* or pre-pub* or preadolescen* or pre-adolescen* or "young adult*" or "young people" or "young person" or "young man" or "young men" or "young woman" or "young women" or student*).ti,ab. 4746518

3 ("mental difficult*" or "mental health challenge*" or "mental health difficult*" or "mental health problem*" or "mental challenge*" or "mental well$being" or "mental health well$being" or "mental illness" or "mental health illness" or "mood disorder*" or "mental health" or "attention deficit disorder" or ADHD or "behavio$r disorder*" or "communication disorder*" or "conduct disorder*" or "emotional adjustment*" or "emotional* disturb*" or "intellectual development disorder*" or "learning disorder*" or "psychiatric patient*" or psychopatholog* or suicid* or "thought disturbance*" or psychotherap* or "child psychopatholog*" or psychiatr* or anxiety or depression or suicid* or stress or psychos* or psychotic* or bipolar* or schizophren* or schizoaff* or "eating disorder*" or bulimi* or anorexi*).ti,ab. 6288586

4 ("cell* phone*" or "mobile phone*" or smartphone* or "smart phone*" or "personal digital assistant*" or PDA or internet* or app or apps or application* or "mobile device*" or "mobile app*" or smartphone* or "mobile phone*" or digital* or technolog* or computer* or mhealth* or mhealth or mobile health or ehealth or "electronic health" or "social media" or phone or online or web-based).ti,ab. 6989448

5 (treatment* or intervention* or therap* or training or support* or help* or assistan* or development or evaluation or trial* or RCT* or evidence or efficacy or effectiveness or implementation or case-control or fidelity or assessment or clinical stud* or testing or acceptability or feasibility or adoption or clinical trial or randomized controlled trial).ti,ab. 38094753

6 limit 1 to yr="2021 -Current" 1653950

7 limit 2 to yr="2021 -Current" 711658

8 limit 3 to yr="2021 -Current" 1011603

9 limit 4 to yr="2021 -Current" 1543529

10 limit 5 to yr="2021 -Current" 6181728

11 6 and 7 and 8 and 9 and 10 8003

**Search results**

Exported all 8003 references to RefWorks

Removed Duplicates (n=855)

Final references (n=7148)

Full-text and abstract screening (n=7148)

Excluded (n=7018)

Full text screening (n=130)

Records excluded (n = 127), records included (n = 3)

| **Section and Topic** | **Item #** | **Checklist item** | **Location where item is reported** |
| --- | --- | --- | --- |
| **TITLE** | | |  |
| Title | 1 | Identify the report as a systematic review. | Page 1 |
| **ABSTRACT** | | |  |
| Abstract | 2 | See the PRISMA 2020 for Abstracts checklist. | Page 1 |
| **INTRODUCTION** | | |  |
| Rationale | 3 | Describe the rationale for the review in the context of existing knowledge. | Page 2-4 |
| Objectives | 4 | Provide an explicit statement of the objective(s) or question(s) the review addresses. | Page 4 |
| **METHODS** | | |  |
| Eligibility criteria | 5 | Specify the inclusion and exclusion criteria for the review and how studies were grouped for the syntheses. | Page 5-6 |
| Information sources | 6 | Specify all databases, registers, websites, organisations, reference lists and other sources searched or consulted to identify studies. Specify the date when each source was last searched or consulted. | Page 5 |
| Search strategy | 7 | Present the full search strategies for all databases, registers and websites, including any filters and limits used. | Page 5 and appendix 1 |
| Selection process | 8 | Specify the methods used to decide whether a study met the inclusion criteria of the review, including how many reviewers screened each record and each report retrieved, whether they worked independently, and if applicable, details of automation tools used in the process. | Page 5 |
| Data collection process | 9 | Specify the methods used to collect data from reports, including how many reviewers collected data from each report, whether they worked independently, any processes for obtaining or confirming data from study investigators, and if applicable, details of automation tools used in the process. | Page 7 |
| Data items | 10a | List and define all outcomes for which data were sought. Specify whether all results that were compatible with each outcome domain in each study were sought (e.g. for all measures, time points, analyses), and if not, the methods used to decide which results to collect. | Page 4-8 |
|  | 10b | List and define all other variables for which data were sought (e.g. participant and intervention characteristics, funding sources). Describe any assumptions made about any missing or unclear information. | Page 4-8 |
| Study risk of bias assessment | 11 | Specify the methods used to assess risk of bias in the included studies, including details of the tool(s) used, how many reviewers assessed each study and whether they worked independently, and if applicable, details of automation tools used in the process. | Page 6 |
| Effect measures | 12 | Specify for each outcome the effect measure(s) (e.g. risk ratio, mean difference) used in the synthesis or presentation of results. | N/A |
| Synthesis methods | 13a | Describe the processes used to decide which studies were eligible for each synthesis (e.g. tabulating the study intervention characteristics and comparing against the planned groups for each synthesis (item #5)). | Page 4-8 |
|  | 13b | Describe any methods required to prepare the data for presentation or synthesis, such as handling of missing summary statistics, or data conversions. | N/A |
|  | 13c | Describe any methods used to tabulate or visually display results of individual studies and syntheses. | N/A |
|  | 13d | Describe any methods used to synthesize results and provide a rationale for the choice(s). If meta-analysis was performed, describe the model(s), method(s) to identify the presence and extent of statistical heterogeneity, and software package(s) used. | N/A |
|  | 13e | Describe any methods used to explore possible causes of heterogeneity among study results (e.g. subgroup analysis, meta-regression). | N/A |
|  | 13f | Describe any sensitivity analyses conducted to assess robustness of the synthesized results. | Page 4-8 |
| Reporting bias assessment | 14 | Describe any methods used to assess risk of bias due to missing results in a synthesis (arising from reporting biases). | Page 4-8 and appendix |
| Certainty assessment | 15 | Describe any methods used to assess certainty (or confidence) in the body of evidence for an outcome. | N/A |
| **RESULTS** | | |  |
| Study selection | 16a | Describe the results of the search and selection process, from the number of records identified in the search to the number of studies included in the review, ideally using a flow diagram. | Page 11 -25 |
|  | 16b | Cite studies that might appear to meet the inclusion criteria, but which were excluded, and explain why they were excluded. | Page 11 -25 |
| Study characteristics | 17 | Cite each included study and present its characteristics. | Page 11 -25 |
| Risk of bias in studies | 18 | Present assessments of risk of bias for each included study. | Page 11 -25 and appendix |
| Results of individual studies | 19 | For all outcomes, present, for each study: (a) summary statistics for each group (where appropriate) and (b) an effect estimate and its precision (e.g. confidence/credible interval), ideally using structured tables or plots. | Page 11 -25 |
| Results of syntheses | 20a | For each synthesis, briefly summarise the characteristics and risk of bias among contributing studies. | Page 11 -25 |
|  | 20b | Present results of all statistical syntheses conducted. If meta-analysis was done, present for each the summary estimate and its precision (e.g. confidence/credible interval) and measures of statistical heterogeneity. If comparing groups, describe the direction of the effect. | Page 11 -25 |
|  | 20c | Present results of all investigations of possible causes of heterogeneity among study results. | Page 11 -25 |
|  | 20d | Present results of all sensitivity analyses conducted to assess the robustness of the synthesized results. | Page 11 -25 |
| Reporting biases | 21 | Present assessments of risk of bias due to missing results (arising from reporting biases) for each synthesis assessed. | Page 11 -25 and limitations |
| Certainty of evidence | 22 | Present assessments of certainty (or confidence) in the body of evidence for each outcome assessed. | Page 11 -25 |
| **DISCUSSION** | | |  |
| Discussion | 23a | Provide a general interpretation of the results in the context of other evidence. | Page 29 - 35 |
|  | 23b | Discuss any limitations of the evidence included in the review. | Page 29 - 35 |
|  | 23c | Discuss any limitations of the review processes used. | Page 29 - 35 |
|  | 23d | Discuss implications of the results for practice, policy, and future research. | Page 29 - 35 |
| **OTHER INFORMATION** | | |  |
| Registration and protocol | 24a | Provide registration information for the review, including register name and registration number, or state that the review was not registered. | Page 1 |
|  | 24b | Indicate where the review protocol can be accessed, or state that a protocol was not prepared. | Page 1 |
|  | 24c | Describe and explain any amendments to information provided at registration or in the protocol. | Provided |
| Support | 25 | Describe sources of financial or non-financial support for the review, and the role of the funders or sponsors in the review. | Provided |
| Competing interests | 26 | Declare any competing interests of review authors. | Provided |
| Availability of data, code and other materials | 27 | Report which of the following are publicly available and where they can be found: template data collection forms; data extracted from included studies; data used for all analyses; analytic code; any other materials used in the review. | Provided |

*From:*  Page MJ, McKenzie JE, Bossuyt PM, Boutron I, Hoffmann TC, Mulrow CD, et al. The PRISMA 2020 statement: an updated guideline for reporting systematic reviews. BMJ 2021;372:n71. doi: 10.1136/bmj.n71
