# Supplementary material for: The Acceptability, Engagement, and Feasibility of Mental Health Apps for Marginalized and Underserved Young People: Systematic Review and Qualitative Study
Source: J Med Internet Res. 2024 Jul 30;26:e48964. doi: 10.2196/48964 (PMC11322694; doi:10.2196/48964)
Supplement: Multimedia Appendix 2 [file jmir_v26i1e48964_app2.docx]

# TOPIC GUIDE

**General questions (Adoption)**

1. How did you first hear about this study? Where did you see the advert?
2. Why did you decide to take part in this research?
3. Do you have any friends or anyone you know who are also taking part?

**Implementation questions**

**Usage**

1. Can you describe whether you have used the app and if so, how often? (**if not, jump to Engagement questions**)
2. What badges have you earned?
3. What motivated you to continue using the app?
   1. How important were the badges to you? Were they important because of payment or another reason?
   2. How important was the questionnaire payment?
   3. How important was the app itself in motivating you?
4. Would you have used the app less frequently if you weren't being paid to use it?

**Acceptability and usability**

*Primarily usability*

1. Can you describe when you normally use the app?
   1. Are there particular times or instances when you tend to use the app?
2. How did you find the app to use in general?
3. Easy to use? (e.g., were you able to find the things you wanted easily?)
4. Did you experience any technical problems?

*Questions specific to the MyMoodCoach app*

1. Do you remember what you first thought of the app when you first downloaded it and used it?
   1. Any features you noticed or that grabbed you initially? Did you like to them/ dislike them?
2. As you’ll know, the app includes a number of different features:

*(the badge system, library, videos, mood monitoring, challenges, if/then, tools, explore, voice recordings).*

1. What features did you use and what features did you not use?
   - 1. Why?
     2. What did you like about them?
     3. What did you dislike about them?
     4. Were any features unusable?
2. Did you use tools and challenges frequently?
   - 1. What stopped you from using these features more?
     2. Is there anything that would increase your use of these features?
3. *Ask about specific features of the app if not brought up by the participant*. How did you find the design and visual aspects of the app? (e.g., colour, font type, font size, spacing, graphics, animations) Do you think the design was appropriate for a person like you?
4. Did you find that the app allowed enough personalization/customisation? (e.g., would have liked the app to allow you to upload pictures, set up a profile, modifying dashboard, etc.?)
5. Would you have liked to interact with other users of the app? (e.g., chatting with other users of the app via a chat or forum, having a buddy system)
6. What did you think about the language or wording of the app?
7. Do you think the kind of language was appropriate for a person like you?
8. Were you ever unsure of what the app was saying?
9. Is there anything that would improve your experience of using the app? (e.g., visuals, type of language, exercises, activities, etc.)

*Primarily acceptability*

1. Did you have any worries about using the app? (e.g., did you feel safe and at ease using the app in terms of confidentiality, cyber-bullying, stigma? Did you feel self-conscious about using the app in public? Did you feel like your information was appropriately safeguarded with the app?)
2. How did you find the information provided by the app? Was it helpful? (e.g., was it clear, concise, relevant, interesting, insightful?)
3. Did you feel like the information provided by the app matched your needs?
4. Were you ever unsure of what the app was asking you to do?
5. Do you think this app is relevant to someone like you? (e.g., did you feel like the app was more targeted towards one particular gender or social group? Do you have any suggestions about how to make the app appealing to other groups of young people?)
   1. Do you think the app is well suited to young men?
   2. What about people who are not students or who may be going through a period of being out of work.
6. Did you feel like the app gave you the amount of control you wanted (e.g., you were allowed to navigate it the way you wanted to?)

**Engagement**

1. If you did not use the app, what stopped you from using the app? If you did use the app, what would have made you use it more? Why didn’t you use it more than you did?
2. What made you pick up the app? What made you put it down?
3. Did you often find yourself stopping activities mid-through? Why?
4. Did you find the app engaging?
5. Do you sometimes want to return to certain parts of the app to remind yourself of something, or to practice or learn something?
6. How easy is this to do?
7. Can you describe an instance when you have done this?

**Outcomes**

1. What do you think the app was trying to help you do?
   1. What have you learned from using the app? Is there anything you know now that you didn’t know before?
2. Can you tell me a bit about the effects you have experienced from using the app? (positive or negative)
   1. Can you give me concrete examples of this? (e.g., has it improved your mood, stress levels, sleep, etc.?)

- Increased knowledge about coping and emotions
- Increased your insight into yourself and your emotions?
- Improved helpful habits?

**If participant reports having experienced negative effects from using the app, ask question 25**

1. Which elements of the app did not work well for you or that been unhelpful?

**Sustainability and payment models**

1. Have you or would you refer any of your friends to this study?
   1. If yes, why?
2. Would you continue to use the app?
3. Would you recommend this app to a friend?
4. Do you use any other health/fitness, well-being, meditation, mindfulness, mood tracking, or mental health apps (e.g., HeadSpace, Calm)? If so, which ones? How does this one compare to other similar apps?
5. Do you think any features of your favourite apps (content or navigation or anything else) would/could have a place in this app?
6. Do you currently pay for any apps?
7. What do you think about in-app purchases?
8. What do you think about advertising in apps?
9. Would you pay for this app?
10. If yes, how much would you pay?
11. What payment model would you prefer?
    - 1. Provide examples of different models (e.g., monthly subscription vs. lifetime subscription)
      2. If someone else paid for it (i.e., your university) would you use it?
12. Who do you think should be paying? Provide examples.
13. Who do you think should make the app available?
14. Where would be the best place to hear about the app?

**Other comments**

1. Are there any other thoughts you would like to share about the app?
